# BARRIERS AND FACILITATORS TO PHYSICAL ACTIVITY AFTER TRANSCATHETER AORTIC VALVE REPLACEMENT: A MIXED-METHODS STUDY

**DOI:** 10.2340/jrm.v57.39974

**Published:** 2025-02-18

**Authors:** Zhiyun SHEN, Xiaojue QIAN, Chenxu HUANG, Daxin ZHOU, Xiaohua XU, Jiaying LV, Ying LIN, Yuxia ZHANG

**Affiliations:** 1 Department of Nursing, Zhongshan Hospital, Fudan University, Shanghai, China; 2 Department of Cardiology, Zhongshan Hospital, Fudan University, Shanghai, China; 3 Analytics, Novartis China, Shanghai, China

**Keywords:** cardiac rehabilitation, exercise, physical activity, transcatheter aortic valve replacement

## Abstract

**Objective:**

To evaluate post-transcatheter aortic valve replacement (TAVR) physical activity and explore the factors influencing participation.

**Design:**

A quantitatively driven sequential explanatory mixed-methods study was performed from October 2021 to February 2022 in Shanghai, China.

**Patients:**

The study sample comprised 195 patients who underwent TAVR (58.46% men, mean age = 74.38 years.

**Methods:**

A cross-sectional survey was conducted to assess the extent of physical activity maintenance after TAVR via the International Physical Activity Questionnaire-Short Form (IPAQ-SF). Preliminary factors were identified via Poisson regression. Subsequently, Fogg’s behaviour model-guided targeted qualitative interviews were conducted to confirm and expand on barriers and facilitators to physical activity engagement.

**Results:**

93.33% of post-TAVR patients lacked regular physical activity. Fourteen barriers and facilitators were identified and grouped into motivation (health expectation, social belonging, feeling after physical activity, kinesiophobia), ability (complex forms of physical activity, misperceptions, scheduling conflicts, traffic and distance, self-regulation), and triggers (surroundings and environment, peer and family support, professional support, mobile health, internalization of exercise habits).

**Conclusion:**

The study findings indicate low adherence to regular physical activity among patients post-TAVR. Intervention strategies that increase patients’ motivation and ability to perform physical activity and provide appropriate triggers should be further developed.

## LAY ABSTRACT

Physical activity is vital for well-being post-transcatheter aortic valve replacement (TAVR), a procedure that offers hope to frail or elderly patients. Despite the importance of physical activity, physical inactivity is common after TAVR, and factors influencing post-TAVR activity remain unstudied. In this mixed-methods study, we evaluated post-TAVR physical activity levels and explored the associated barriers and facilitators. The findings indicate that a majority (93.33%) of patients lack regular physical activity, highlighting the need to monitor patient rehabilitation. Identified physical activity barriers and facilitators at the patient level can be used to inform future measures to promote post-TAVR physical activity. Addressing these factors may not only enhance rehabilitation but may also contribute to a better quality of life for TAVR patients.

Transcatheter aortic valve replacement (TAVR) is a novel treatment that has become a standard procedure for aortic stenosis, especially for older adults at risk of poor outcomes with open cardiac surgery (1, 2). With the rapid development of TAVR over the past 2 decades, the 2-year survival rate of patients with severe symptomatic aortic stenosis has greatly improved from 50% to above 95% (1, 3, 4). However, beyond avoiding and troubleshooting procedural complications, it is also essential to further identify what functional status patients should expect to achieve after successful and uneventful TAVR (5). Researchers have reported that although survival time has improved, reduced exercise capacity, physical inactivity, and frailty still persist and contribute to a greater risk of mortality and functional decline following TAVR (6–8). Among these problems, physical inactivity has raised much concern and was found to be predictive of 12-month mortality and worsening disability after TAVR (7).

Physical activity, defined as any bodily movement produced by skeletal muscles resulting in energy expenditure beyond resting expenditure (9), has been confirmed to improve exercise capacity and frailty in patients after TAVR (10–13). In recent years, there has been a growing focus among guidelines and expert consensuses on emphasizing the importance of physical activity for patients after TAVR (13–17). Although the importance of physical activity has been well validated, poor adherence to physical activity recommendations is a significant problem among patients after TAVR (16, 18). One study including individuals from Canada, France, and America revealed that half of surviving patients experienced a paradoxical decline in physical activity following TAVR (7). Efficient strategies promoting physical activity are urgently needed for patients with TAVR to achieve sustainable changes in exercise capacity and long-term outcomes. Determining the barriers and facilitators to participation in physical activity is therefore a necessary step before developing strategies to determine the emphasis and priority of interventions.

Previous studies have extensively explored the barriers and facilitators to participating in physical activity during cardiac rehabilitation among individuals with heart failure or coronary heart disease (19, 20). These studies have revealed a wide range of factors, including personal level, socioenvironmental level, and intervention-related level (21). Barriers and facilitators to health behaviours tend to be disease-specific (22, 23). As a new technology, TAVR is mostly used to treat extreme, high, and intermediate surgical risk patients with symptomatic severe aortic stenosis (3). Considering the potential differences in changes in physical function and psychological status between patients after TAVR and those after other cardiovascular procedures, it is reasonable to hypothesize that the barriers and facilitators to participation in physical activity for the post-TAVR patient population may differ from those reported in the literature. However, few studies have focused on the barriers and facilitators to physical activity, especially in the population undergoing TAVR. The factors influencing patients’ engagement in physical activity after TAVR remain unclear.

The aim of this study was to systematically evaluate the levels of post-TAVR physical activity and to identify the key determinants influencing patient engagement in such activities. To answer this complex research question, we used a sequential explanatory mixed-methods study design (24). We first collected quantitative data to provide an overall picture of the research question and then collected qualitative data to explain the quantitative findings. With this type of research design, we could gain a deep understanding of the phenomenon under study.

## Methods

### Design

**Fig. 1:**
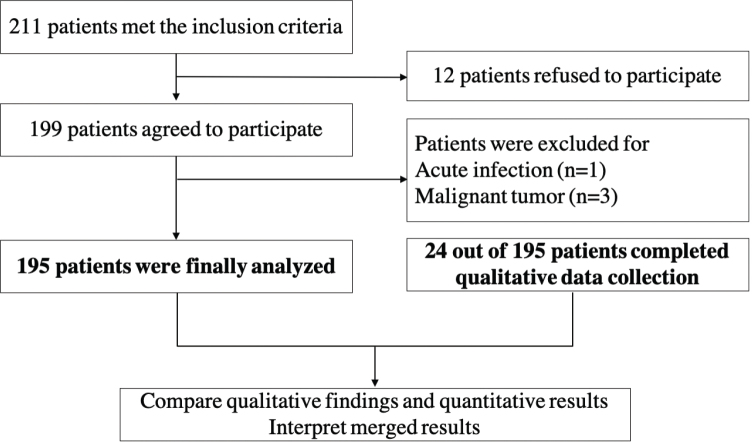
Participant flow diagram.

A quantitatively driven sequential explanatory mixed-methods study was conducted, comprising a cross-sectional survey and qualitative face-to-face interviews (Fig. 1). The cross-sectional survey assessed the extent of physical activity maintenance after TAVR and identified preliminary factors influencing physical activity. Additionally, the survey determined which patients actively engaged in physical activity and which did not. Targeted qualitative interviews were then conducted to identify the barriers and facilitators to physical activity engagement.

The study was designed and implemented with due consideration of the methodological assumptions, principles, and practices underpinning mixed-methods studies (25). The report was prepared following Good Reporting of a Mixed Methods Study (GRAMMS) guidelines (26) (see Table SI).

### Quantitative methods

*Participants*. The study was conducted at a tertiary teaching hospital in Shanghai, China. We collected data from consecutive patients who underwent outpatient review after TAVR from October 2021 to February 2022. Patients 3 months or more post-TAVR who could understand and communicate in Chinese and who had sufficient hearing and vision for compliance with the assessments were included. Patients who were unwilling to participate in this study, who had other serious diseases (acute infection, malignant tumour, acute cerebrovascular diseases, or terminal illness), or who had severe mental disorders were excluded. A total of 195 patients were included in the cross-sectional survey.

*Sample size.* A single population proportion formula was used to determine the sample size on the basis of the following assumptions. The proportion of physical activity was 27% (7), with a 95% Cl, 8% margin of error (d), and 20% nonresponse rate, resulting in a necessary sample size of at least 148 participants. To obtain robust and reliable results, we ultimately included as many participants as possible. During the study period, a total of 211 inpatients were eligible to participate in the study; 12 patients refused to participate (5.69%), and 4 patients were excluded for acute infection or malignant tumours (1.90%). Finally, 195 patients (92.42%) were included.

### Measures

*Physical activity.* The validated Chinese version of the International Physical Activity Questionnaire-Short Form (IPAQ-SF) was used to measure self-reported physical activity levels (27, 28). The IPAQ-SF is used to gather data on the frequency and duration of walking, moderate-intensity activities, vigorous-intensity activities, and sitting over the past 7 days. This information is then utilized to calculate energy expenditure in metabolic equivalents (METs). The continuous score of the IPAQ-SF is computed by multiplying the MET level by the minutes of activity per day and the days per week, resulting in a measurement expressed in METs-min/week. This calculation applies to walking (3.3 METs), moderate physical activity (4 METs), and vigorous physical activity (8 METs) (29). The categorical score of the IPAQ-SF categorizes a patient’s physical activity (PA) level as “low”, “moderate”, or “high”. These classifications can be further interpreted as “physically active” (group A, corresponding to “moderate” or “high” PA levels) and “physically inactive” (group B, associated with a “low” PA level) (refer to Appendix S1 for details).

*Influencing factors of physical activity.* On the basis of both literature analysis and clinical experience, we developed a comprehensive questionnaire focused on influential factors. This questionnaire is used to assess demographics, lifestyle factors, support from healthcare professionals, medical variables, laboratory data, and self-reported outcomes (such as dizziness, headache, painful joints, painful muscles, painful wounds, dyspnoea, fatigue, chest tightness, and palpitations) experienced after TAVR.

*Data collection.* The research team contacted TAVR patients from the outpatient department and invited eligible participants on the basis of the inclusion and exclusion criteria. Potential participants received comprehensive information concerning the research, including benefits and risks, and were made aware of their right to withdraw without affecting their medical treatment. Written informed consent was obtained. Assessment instruments were administered by trained researchers, who encouraged the autonomous completion of questionnaires. For participants facing literacy challenges, researchers read aloud, ensuring a thorough understanding. Additional factors influencing physical activity, such as medical variables and laboratory data, were collected by trained researchers through medical record reviews.

*Statistical analysis*. Quantitative analysis was conducted via SPSS v26.0 (IBM *Corp*, *Armonk,* NY, *USA*). Multiple imputations with chain equations were used for missing values. Data are presented as the means and standard deviations (SDs) for continuous variables and as percentages for categorical variables. Independent-samples *t-*tests were used to compare normally distributed continuous variables, whereas the Mann–Whitney *U* test was used to analyse non-normally distributed continuous variables. Categorical variables were assessed via the Pearson χ^2^ test, the Pearson χ^2^ test with Yates’ continuity correction, and Fisher’s exact test. Poisson regression was used to examine univariable and multivariable factors for PA, with *p*-values <0.2 considered for multivariable analysis. The results of the Poisson regression are reported as incidence rate ratios (IRRs) with corresponding 95% confidence intervals (CIs); *p* < 0.05 was considered to indicate statistical significance.

### Qualitative method

The qualitative approach involved a qualitative descriptive design to offer straightforward descriptions of the barriers and facilitators to physical activity post-TAVR. With this method, we collected qualitative data to elucidate and further enrich the quantitative findings.

*Participants.* Following the physical activity survey, we strategically used purposive sampling, integrating typical case sampling and maximum variation sampling (30), to assemble a diverse cohort for our study on physical activity post-TAVR. This method allowed us to include participants with a continuum of physical activity levels, from highly active to sedentary, as well as a variety of patient characteristics, such as age, sex, education level, postoperative duration, and living conditions, thereby enriching the representativeness of our study population. By conducting face-to-face surveys, we were able to evaluate patients’ communicative and expressive capabilities and compile comprehensive data on their physical activity levels and demographic details. The principal investigator classified participants into active and inactive categories on the basis of survey responses, which aided in the selective recruitment of a diverse patient cohort. With the aim of maximizing sample diversity, we deliberately recorded each patient’s characteristics, ensuring that each new participant’s profile significantly diverged from the previous profile. This systematic sampling continued until thematic saturation was attained, marked by the point at which no new themes or codes emerged from the qualitative data analysis. Saturation was initially noted after the 21st interview, but to ensure the robustness of our qualitative findings we elected to include 3 more participants. By the end of our data collection, we had interviewed 24 patients who had provided informed consent. This deliberate sampling approach, confirmed by thematic saturation, ensured that a representative sample across key variables was essential for our study. This methodical strategy resulted in a rich dataset, enabling an in-depth analysis of factors influencing physical activity post-TAVR, aligning with the qualitative descriptive scope of our research.

*Interviews.* Potential participants were invited to undergo face-to-face interviews following the survey in a quiet meeting room in the hospital. A semi-structured interview outline was designed on the basis of Fogg’s behaviour model by the research team (see Appendix S2), which had been pilot tested. The example questions were as follows: “How have you performed physical activity since your discharge?”, “What motivates you to persist (not persist) in participating in physical activity?”, “Why can (can’t) you maintain physical activity?”, “What will trigger you to stay in physical activity?”, “What will hinder you from maintaining physical activity?”, “How do you overcome and solve these barriers during your physical activity?”, and “What help do you think you need from the outside to sustain physical activity?”. The duration of the face-to-face interviews was 30–60 min. Participants were encouraged to discuss their experiences with physical activity after TAVR and the barriers and facilitators to participation. The first author, a female registered nurse experienced in cardiac rehabilitation for TAVR patients, conducted all 24 interviews. An intelligent recording device (iFLYTEK, SR702; https://www.iflytek.com/en/products/recorder/smart-recorder.html) was used, and the audio files were transcribed automatically. The transcripts were carefully checked and revised verbatim by 2 researchers within 24 h after the interview.

*Data analysis.* NVivo version 12 (https://lumivero.com/) was used to store and analyse the qualitative data. Data collection and analysis were conducted simultaneously. The conventional deductive content analysis (31) was used following a 4-step iterative approach: (1) extracting codes from excerpts, (2) conceptualizing the codes to initial concepts, (3) analysing the relationships between concepts and connecting them into categories through deduction and induction, and (4) integrating and refining categories to generate a theme. Finally, the categories with each theme were mapped into Fogg’s behaviour model according to the explanations and examples of motivation, ability, and triggers of behaviours proposed by Fogg (32). To bolster the reliability of our findings, 2 researchers (ZS and XQ) independently coded the data, with a third researcher (YZ) resolving any coding differences through discussion. We also sought to mitigate biases by involving 4 nursing experts from Fudan University in a peer debriefing process, which included onsite presentations and feedback on our methodology, interpretations, and themes. This collaborative approach helped to validate our research outcomes and refine our analytical process.

### Data integration

Contiguous and narrative approaches were used for data integration. The results are presented separately through the contiguous approach (33). According to the order in which the development of the study took place, quantitative results are presented first, and qualitative results are presented second (34). The narrative approach was used to present a discussion of the overall results in the Discussion section and in the fourth Table (35). The results of both phases are combined in the Discussion section and the fourth Table to answer the research questions. The quantitative results help determine the extent of physical activity maintenance after TAVR and identify primary barriers and facilitators to physical activity participation. The qualitative results provide an in-depth exploration of barriers and facilitators to physical activity participation after TAVR. This process allows the qualitative results to clarify and explain the statistical findings of the quantitative phase.

## Results

### Participant characteristics

A total of 195 participants were included in this study. The mean participant age was 74.38 (SD 6.06) years, and 58.46% were males. The participants were divided into 2 groups according to whether they had appropriate physical activity. The demographic characteristics of the physical activity group (Group A) and physical inactivity group (Group B) are presented in Table I. The participants who were physically active were younger. Compared with those who maintained physical inactivity, those who were physically active had higher literacy levels, higher physical activity frequency before TAVR, more support from health professionals after discharge, shorter durations of aortic stenosis, and less self-reported discomfort after TAVR.

**Table I:** Demographic characteristics and lifestyle factors associated with physical activity post-TAVR

Variables	Total (*n* =195)	Group A (*n* =13)	Group B (*n* =182)	*p-*value
Demographic characteristics				
Age (years), mean (SD)	74.38 (6.06)	71.23 (7.77)	74.60 (5.89)	0.097^1^
Male, *n* (%)	114 (58.46)	9 (68.23)	105 (57.69)	0.415^2^
Living alone, *n* (%)	18 (9.23)	0 (0.00)	18 (9.89)	0.488^3^
Living in rural area, *n* (%)	60 (30.77)	1 (7.69)	59 (32.42)	0.120^3^
Married/cohabitating, *n* (%)	165 (84.62)	13 (100.00)	152 (83.52)	0.233^3^
College-educated, *n* (%)	34 (17.44)	5 (38.46)	29 (15.93)	0.091^3^
Lifestyle factors				
Smoking history, *n* (%)				0.137^4^
Never smoked	142 (72.82)	8 (61.54)	134 (73.63)	
Gave up smoking for more than 1 month	50 (25.64)	4 (30.77)	46 (25.27)	
Currently smoking, within 1 month	3 (1.54)	1 (7.69)	2 (1.10)	
PA frequency ≥3 times/week before TAVR, *n* (%)	21 (10.77)	7 (53.85)	168 (76.92)	< 0.001^3^
Received support for physical activity from healthcare professionals, *n* (%)				
Had ever participated in a physical activity programme	17 (8.72)	3 (23.08)	14 (7.69)	0.164^3^
Received support during hospitalization	52 (26.67)	5 (38.46)	47 (25.82)	0.502^3^
Received support after discharge	23 (11.79)	6 (46.15)	17 (9.34)	< 0.001^3^
Medical variables				
Perioperative complications, *n* (%)	33 (16.92)	1 (7.69)	32 (17.58)	0.592^3^
Prior PCI, *n* (%)	24 (12.31)	0 (0.00)	24 (13.19)	0.336^3^
Polypharmacy, *n* (%)	82 (42.05)	5 (38.46)	77 (42.31)	0.786^2^
Multimorbidity, *n* (%)	174 (89.23)	11 (84.62)	163 (89.56)	0.926^3^
Years with AS, mean (SD)	7.57 (9.76)	3.92 (3.77)	7.83 (10.01)	0.023^1^
Months post-TAVR, mean (SD)	24.19 (17.39)	22.23 (16.15)	24.33 (17.50)	0.815^1^
Hospital LOS, days, mean (SD)	11.81 (6.16)	11.15 (3.31)	11.86 (6.31)	0.814^1^
BMI (kg/m^2^), mean (SD)	23.83 (3.40)	24.18 (4.06)	23.81 (3.36)	0.708^5^
NYHA class, *n* (%)				
I	3 (1.54)	1 (7.69)	2 (1.10)	0.270^4^
II	17 (8.72)	1 (7.69)	16 (8.79)	
III	139 (71.28)	8 (61.54)	131 (71.98)	
IV	36 (18.46)	3 (23.08)	33 (18.13)	
LVEF before TAVR (%), mean (SD)	57.72 (12.16)	57.77 (11.80)	57.71 (12.21)	0.961^1^
LVEF at discharge (%), mean (SD)	58.62 (10.72)	56.85 (14.00)	58.74 (10.48)	0.945^1^
LVEF one-month post-TAVR (%), mean (SD)	60.05 (9.29)	61.23 (12.54)	59.97 (9.06)	0.218^1^
Laboratory data				
Hb before TAVR (g/L), mean (SD)	128.29 (18.69)	130.69 (17.42)	128.12 (18.81)	0.358^1^
Hb post-TAVR (g/L), mean (SD)	112.65 (18.05)	119.15 (16.61)	112.19 (18.10)	0.191^1^
GFR at baseline before TAVR (ml/min/1.73 m^2^), mean (SD)	69.56 (19.12)	71.31 (16.66)	69.44 (19.31)	0.925^1^
GFR at baseline post-TAVR (ml/min/1.73 m^2^), mean (SD)	74.88 (19.26)	78.38 (9.99)	74.63 (19.74)	0.980^1^
Cr before TAVR (μmol/L), mean (SD)	99.46 (98.40)	90.08 (25.47)	100.13 (101.63)	0.600^1^
Cr post-TAVR (μmol/L), mean (SD)	92.04 (88.16)	80.62 (12.00)	92.85 (96.16)	0.636^1^
cTnT before TAVR (ug/L), mean (SD)	0.08 (0.45)	0.02 (0.01)	0.09 (0.47)	0.101^1^
cTnT post-TAVR (ug/L), mean (SD)	0.36 (0.93)	0.26 (0.15)	0.37 (0.76)	0.712^1^
Self-reported discomfort post-TAVR	94 (48.21)	1 (7.69)	93 (51.10)	0.002^2^

AS: aortic stenosis; BMI: body mass index; Cr: creatinine; cTnT: cardiac troponin T; GFR: glomerular filtration rate; Hb: haemoglobin; LOS: length of stay; LVEF: left ventricular ejection fraction; NYHA: New York Heart Association; PCI: percutaneous coronary intervention; TAVR: transcatheter aortic valve replacement.

1 Mann–Whitney *U* test;

2 Pearson χ^2^ test;

3 Pearson χ^2^ test with Yates’ continuity correction;

4 Fisher’s exact test;

5 independent-samples *t*-test.

### Physical activity

Only 6.67% (13/195) of the participants who underwent TAVR had appropriate physical activity levels. A total of 93.33% (182/195) of the participants still maintained physical inactivity after TAVR.

### Determinants of physical activity

The incidence of physical activity was very low in this study (6.67%), and the data conformed to the Poisson distribution. Thus, the Poisson regression model explored the multivariable factors for physical activity levels. The results are presented in Table II. In the multivariate analysis, physical activity frequency (≥3 times/week) before TAVR (IRR 6.933, 95% CI 1.385–34.711, *p*=0.018), having received physical activity support from health professionals after discharge (IRR 8.395, 95% CI 1.349–52.243, *p*=0.023), and self-reported discomfort after TAVR (IRR 0.093, 95% CI 0.010–0.885, *p*=0.039) were found to be independent factors for predicting physical activity status among participants after TAVR.

**Table II:** Factors associated with physical activity status post-TAVR identified via Poisson regression analysis (*n*=195)

Variables	IRR	95% CI	*p*-value
Physical activity frequency ≥3 times/week before TAVR	6.933	1.385–34.711	0.018
Have received support after discharge	8.395	1.349–52.243	0.023
Self-reported discomfort post-TAVR	0.093	0.010–0.885	0.039

CI: confidence interval; IRR: incidence-rate ratio; TAVR: transcatheter aortic valve replacement. In the Poisson regression analysis: p-values <0.2 were included in the multivariable analysis. The data aligned well with the Poisson distribution: as indicated by the Pearson χ² test: which yielded a χ² statistic of 0.0588 and a corresponding p-value of 0.8084. The results of the Poisson regression analysis are presented as IRRs with the corresponding 95% CIs.

### Barriers and facilitators to participation in physical activity

**Fig. 2:**
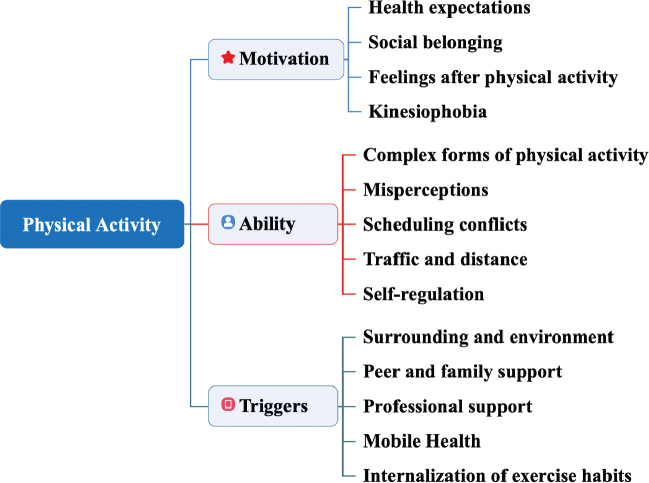
Barriers and facilitators to physical activity after transcatheter aortic valve replacement.

Among the 195 participants, 24 underwent interviews to explore the barriers and facilitators to physical activity participation after TAVR. Both participants who maintained recommended physical activity and those with physical inactivity were included to ensure representation. Three main themes emerged, encompassing 14 categories that were further classified into the themes of motivation, ability, and triggers (Fig. 2). Sample codes, initial concepts, and categories and their relationships to Fogg’s behaviour model are provided in Table SII. A summary of the participants’ clinical and demographic data is provided in Table III.

**Table III:** Demographic and clinical characteristics of the participants in the semi-structured interviews

No.	Sex	Age (years)	College-educated	Postoperative months (months)	Living in a rural area	Group A
P1	Male	60~70	NO	17	No	No
P2	Male	60~70	YES	35	No	No
P3	Male	60~70	NO	17	No	Yes
P4	Female	70~80	YES	18	No	Yes
P5	Female	60~70	NO	29	No	No
P6	Male	60~70	NO	47	No	No
P7	Male	60~70	NO	9	No	Yes
P8	Male	70~80	NO	6	Yes	No
P9	Male	50~60	NO	11	Yes	Yes
P10	Female	70~80	YES	28	No	Yes
P11	Female	70~80	NO	63	No	Yes
P12	Female	60~70	NO	19	No	No
P13	Male	70~80	YES	11	No	No
P14	Male	70~80	NO	42	No	Yes
P15	Female	60~70	NO	20	No	No
P16	Male	80~90	NO	34	No	Yes
P17	Male	60~70	YES	12	No	Yes
P18	Female	70~80	NO	23	No	Yes
P19	Female	70~80	NO	4	Yes	No
P20	Male	70~80	NO	11	No	No
P21	Male	60~70	NO	10	No	No
P22	Female	70~80	NO	13	No	No
P23	Male	60~70	NO	16	No	Yes
P24	Male	70~80	YES	6	No	Yes

### Theme 1: Motivation

*Health expectations.* The benefits of physical activity have been widely publicized. After TAVR, participants who chose to maintain recommended physical activity levels believed that physical activity could make them healthier and help them return to everyday life.

Restoring health is the main objective for me. For my health and to recover better, I must do some regular physical activity. People who do not exercise regularly can’t have a healthier body and return to normal life. (P23)

*Social belonging.* On the one hand, engaging in physical activity can offer participants valuable opportunities to participate in group activities after TAVR. Through these activities, they can interact, share experiences, and support each other. On the other hand, some participants may feel pressured to conform to the wishes of their peers (family members or neighbours), leading them to avoid physical activity and prioritize excessive rest. Such participants may seek validation from their loved ones or neighbours by adhering to their recommendations.

I like to do physical activity in the park so I can meet many old people and communicate with them. (P14)

Sometimes I go out for a walk in the morning, but my neighbours always say I am not in good health and should not go out frequently. If I want to buy something, they ask my son to buy something for me. My son also doesn’t want me to walk for a long time. I feel pressured to conform to their advice. (P21)

*Feelings after physical activity.* Positive feelings after physical activity, such as feeling relaxed and improvement in symptoms, are important reasons why participants persist in participating in physical activity after TAVR. However, discomfort after physical activity is an essential obstacle to persisting in physical activity for participants after TAVR. Some participants experienced pain, palpitations, or poor breathing early during physical activity. Because of this discomfort, they were prone to give up physical activity.

I feel relaxed after walking. If I don’t exercise today, I will feel uncomfortable all over. I insist on proper physical activity so that I can feel more comfortable. (P18)

I can’t maintain physical activity now because when I get up in the morning, my feet zseem to be very sore. Then my heart beats fast when I move. It’s uncomfortable, and I have to block it out. But when I went to the hospital for examination, the doctor said my heart function was OK and I could walk fast. But every time I walk, my heart beats fast, and I feel uncomfortable. I don’t want to walk. (P5)

*Kinesiophobia.* Kinesiophobia is an excessive, irrational, and debilitating fear of movement or physical activity. Participants with severe aortic stenosis have contraindications to exercise before TAVR and are told by medical staff not to exercise. Therefore, participants often develop kinesiophobia. Thus, even if the cardiac function of participants improves after TAVR, they still refuse physical activity because of kinesiophobia. These participants might also worry that the artificial valve will shift.

I am fearful that my valve will fall off or shift after exercise. (P19)

### Theme 2: Ability

*Complex forms of physical activity.* Some interviewees thought it was difficult for participants to learn the steps of physical activity, such as Tai Chi or rehabilitation gymnastics, provided by the medical staff. Therefore, they could not adhere to the physical activity recommended by the medical staff.

When I went to the cardiac rehabilitation centre, the doctor gave me a prescription for physical activity. It was a rehabilitation gymnastics programme named Baduanjin. It was challenging for me to learn and remember the steps of Baduanjin. (P8)

*Misperceptions.* Exercise is a subset of physical activity that is planned, structured, and repetitive and has as a final or an intermediate objective the improvement or maintenance of physical fitness. However, after TAVR, most participants had misconceptions about exercise, physical activity, and daily activities. They regarded daily activities as exercise or physical activity. Therefore, instead of performing regular exercise, they performed daily activities, which cannot meet the recommended amount of physical activity or exercise.

I do exercise. For example, I can cook some food for my husband and son. I also go to the neighbours’ house to talk with them. These activities are also exercise or physical activity. I think it’s enough for me. (P15)

*Scheduling conflicts.* Being busy with housework or social activities is a primary barrier to participants’ exercise plans. The participants in both groups said that some housework or going to parties often made them put aside their original exercise plans.

If there is a friend gathering, my exercise plan today will be put on hold. (P13)

I do all the housework at home. Sometimes I have no time. (P12)

*Traffic and distance.* It was difficult for participants to visit the hospital regularly for exercise-based cardiac rehabilitation because of the long distance and inconvenient transportation. Especially in China, TAVR can be performed at only a few hospitals, and participants come from all over the province. Therefore, after discharge, traffic and distance were the main barriers that affected whether participants continued to visit the hospital for exercise-based cardiac rehabilitation.

Doctors and nurses asked me to visit the hospital for the assessment of cardiac rehabilitation. However, the distance was too far, and I gave up. (P15)

*Self-regulation.* This study highlights participant self-regulation as a primary factor in promoting and sustaining physical activity. After TAVR, participants often still face the challenges of advanced age, frailty, and multiple comorbidities, which can hinder quick recovery. Some individuals might consider abandoning physical activity altogether when faced with intensity beyond their capacity during the early stages. However, there were also participants who gradually increased their physical activity levels on the basis of their individual conditions, ultimately achieving satisfying results. During the interviews, it became evident that many participants who successfully adhered to physical activity had a strong sense of self-regulation.

After the operation, I have no strength and can’t walk independently. I know it’s useless to take any medicine. I think about it. One is to eat well, the other is to walk with the control of heart rate. I bring a stool. If the heart rate is below 80, I keep walking slowly. If the heart rate is above 80, I will sit down. This little bit daily, I gradually increased the amount of exercise. It has taken almost a year. Now I can go out twice a day. Go to the park once in the morning, return, and go out once in the afternoon. (P16)

### Theme 3: Triggers

*Surroundings and environment.* Weather conditions had a noteworthy influence on participants’ physical activity levels. Unfavourable weather tended to discourage participants from engaging in physical activity. Conversely, on sunny days, participants were more inclined to engage in activities such as walking outdoors. Additionally, we observed that participants were more likely to adhere to their exercise routines if their homes or communities provided suitable exercise facilities. The presence of amenities such as sports grounds, distance markers, fitness equipment, and motivational pictures in or near their homes naturally encouraged participants to partake in physical activity.

If it’s rainy today, I won’t go out. If it’s a good day, I will go out for a walk. (P22)

There is a distance marker in our living room. I will count by myself and walk back and forth when I see it. (P14)

*Professional support.* Almost all the participants expressed a lack of professional advice on physical activity after TAVR. Some participants noted that when a doctor or a nurse informed and guided them to perform physical activity during outpatient re-examination or telephone follow-up, they persisted in participating in physical activity.

I am willing to listen to the doctor’s or nurse’s advice, and I am willing to learn because the doctor and nurse are more professional. I don’t listen to the fitness trainer, but there is no doctor or nurse to guide me after discharge. (P15)

After being discharged, I have been consistently guided by a nurse, who enables me to maintain a positive exercise routine. She is very patient and provides excellent guidance. (P14)

*Mobile health.* Checking the number of steps taken was an important trigger in promoting participants’ physical activity. Although all the TAVR participants were older adults, many used smartphones as triggers to perform physical activity by checking parameters, such as the number of steps taken on their smartphones or sports watches.

I will watch the number of steps on my mobile phone. If I can’t reach the goals, I set for myself, I will go right away. (P10)

*Peer and family support.* The participants emphasized the importance of reminders from family members and neighbours in promoting exercise. Peers and families played a crucial role in reminding them to engage in physical activity when they forgot. They also provided encouragement and accompanied them during walks when the participants were reluctant. Furthermore, observing that peers and families maintain regular levels of physical activity served as inspiration for participants to do the same.

If I don’t want to exercise sometimes, my wife and children remind me. We also have a WeChat group, where people often remind me to go out for exercise, such as shadowboxing, walking, or doing something. (P23)

*Internalization of exercise habits.* Participants who maintained regular physical activity highlighted the significance of their pre-existing habits in sustaining motivation post-TAVR. Internalized exercise routines were evident, with some individuals associating walking with a post-dinner tradition, whereas others seamlessly integrated aerobic exercises into their morning rituals. These participants exhibited a strong sense of personal planning and adaptability concerning their physical activity, contributing to their continued engagement in exercise.

I have had the habit of exercising since I was a child. I used to do a lot of exercises before, including table tennis, badminton, treadmill, dancing, and playing tai chi circles. (P17)

## DISCUSSION

As a core component of cardiac rehabilitation, physical activity has been preliminarily confirmed to be beneficial and effective in improving exercise capacity, frailty, and quality of life in patients after TAVR (10–12). However, the present study revealed that only a few patients (6.67%) maintained the recommended physical activity levels after TAVR. Physical inactivity is more common in patients after TAVR than in those after myocardial infarction (36). As a new technology, TAVR is mostly used to treat extreme, high, and intermediate surgical risk patients with symptomatic severe aortic stenosis (3). Historically, physical activity has not been encouraged in patients with severe aortic stenosis before surgery for fear of provoking exertional symptoms such as syncope. However, although a successful TAVR procedure corrects aortic stenosis and enables patients to engage in common physical activities, their behaviour remains closely connected to their individual condition and clinical context. Consistent findings have been reported in previous studies. A study by Sathananthan et al. (7) indicated that TAVR alone may not be sufficient to reverse physical inactivity levels in sedentary individuals. Moreover, a cohort study revealed that even after the successful correction of aortic stenosis through TAVR, 73% of patients performed <150 min/week of moderate or vigorous habitual physical activity, and the mean amount of habitual physical activity decreased at 12 months (7). Nevertheless, the incidence of physical inactivity in our study was higher than that reported in previous studies (7). The possible reason is that the previous study followed patients within 12 months after TAVR longitudinally. We followed patients for a longer duration after TAVR so that their habitual physical activity declined even more.

Although patient recall and self-reports are subjective and prone to information bias, a practical reference standard for quantifying physical activity remains elusive. Accelerometry may be considered an objective evaluation. However, poor accuracy in patients who stroll or wear accelerometers intermittently can also lead to information bias (37). Six-minute walks are objective, but they elicit the patient’s exercise capacity, not their actual behaviour. Questionnaires such as the one used in this study produce self-reported behaviour and can reasonably approximate actual behaviour. A study of 755 patients after TAVR also used patient recall and self-reports to assess their performance of habitual physical activity (7).

Habitual physical activity before TAVR, physical activity support from health professionals after discharge, and discomfort after TAVR are independent predictors of physical activity. A previous study reported that sedentary patients after TAVR were more likely to be older, female, frail, cognitively impaired, depressed, and have multimorbidity (7). Therefore, our results enrich the factors found in previous studies. Moreover, we found that these factors are modifiable. Patients with habitual physical activity before TAVR were more likely to maintain physical activity after TAVR. The qualitative results also confirmed that the internalization of exercise habits facilitated participation in physical activity after TAVR (see Table IV). Medical staff should assess the preoperative physical activity habits of patients (5). For patients without physical activity habits, medical staff should determine methods to motivate patients to perform routine physical activity after TAVR. Unfortunately, the results also revealed that, post-TVAR, most patients never received support for physical activity from health professionals after discharge. Unlike other countries, China has few post-acute care or cardiac rehabilitation centres. After discharge, patients return to their families and communities. However, their rehabilitation after discharge is still in progress, and professional support from medical staff is needed. The medical staff in tertiary hospitals are often too busy to provide patients with advice and continuous care after discharge. Almost all patients expressed a lack of professional support for exercise after TAVR. This phenomenon warrants attention and further intervention. Providing continuous advice on physical activity by medical staff is important for maintaining physical activity among patients after TAVR. There is considerable interest in technology-facilitated home-based cardiac rehabilitation, which may improve physical activity adherence in patients after TAVR (38–40). Telerehabilitation may be used to improve psychological and physical functioning via various technologies and telecommunication strategies (41). Recently, studies have reported that the use of technology tools is growing fast in the cardiac rehabilitation era and promotes exercise-based interventions in a more home-based setting (42). Wearable-assisted home-based cardiac rehabilitation has the potential to act as an adjunct or alternative to centre-based cardiac rehabilitation. Through interviews, we also found that checking on smartphones the number of steps taken is a vital trigger to promote physical activity. In addition, forms of discomfort, such as dizziness, headache, pain, dyspnoea, fatigue, chest tightness, and palpitations, are barriers to physical activity. This finding is consistent with previous research reports (43). These patients maintain physical inactivity for fear that physical activity will lead to further discomfort. Cardiac function improves after TAVR, and patients can tolerate light and moderate physical activity. Furthermore, physical activity can also decrease symptoms of discomfort (12). During the interviews, it was found that kinesiophobia is a barrier to physical activity. Patients with kinesiophobia can be encouraged to start with low levels and minor ranges of physical activity through motivational interviews to allow them to adapt slowly (13).

**Table IV:** Comparison of results and outcomes for the barriers and facilitators to physical activity after TAVR

Barriers & facilitators	Quantitative results	Qualitative results	Outcomes
Motivation	None	Health expectations: Restoring health is the main objective for me. For my health and to recover better, I must do some regular physical activity. People who do not exercise regularly can’t have a healthier body and return to normal life. (P23)	Expansion
	None	Social belonging: I like to do physical activity in the park, so I can meet many old people and communicate with them. (P14) Sometimes I go out for a walk in the morning, but my neighbours always say I am not in good health and should not go out frequently. If I want to buy something, they ask my son to buy something for me. My son also doesn’t want me to go out walking for a long time. I feel pressured to conform to their advice. (P21)	Expansion
	Patients who reported discomfort post-TAVR (IRR 0.093, 95% CI 0.010–0.885, *p*=0.039) were less likely to continue maintaining recommended physical activity levels post-TAVR	Feelings after physical activity: I can’t maintain physical activity now because when I get up in the morning, my feet seem to be very sore. Then, my heart beats fast when I move. It’s uncomfortable, and I have to block it out. But when I went to the hospital for examination, the doctor said my heart function was OK and I could walk fast. But every time I walk, my heart beats fast, and I feel uncomfortable. I don’t want to walk. (P5) I feel relaxed after walking. If I don’t exercise today, I will feel uncomfortable all over. I insist on proper physical activity so that I can feel more comfortable. (P18)	Confirmation/Expansion
	None	Kinesiophobia: I am fearful that my valve will fall off or shift after exercise. (P19)	Expansion
Ability	None	Complex forms of physical activity: When I went to the cardiac rehabilitation centre, the doctor gave me a prescription for physical activity. It was a rehabilitation gymnastics programme named Baduanjin. It was challenging for me to learn and remember the steps of Baduanjin. (P8)	Expansion
	None	Misperceptions: I do exercise. For example, I can cook some food for my husband and son. I also go to the neighbour’s house to talk with them. These activities are also exercise or physical activity. I think it’s enough for me. (P15)	Expansion
	None	Scheduling conflicts: If there is a friend gathering, my exercise plan today will be put on hold. (P13) I do all the housework at home. Sometimes I have no time. (P12)	Expansion
	None	Traffic and distance: Doctors and nurses asked me to go to the hospital for the assessment of cardiac rehabilitation. But the distance was too far, and I gave up. (P15)	Expansion
	None	Self-regulation: After the operation, I have no strength and can’t walk independently. I know it’s useless to take any medicine. I think about it. One is to eat well, the other is to walk with the control of heart rate. I bring a stool. If the heart rate is below 80, I keep walking slowly. If the heart rate is above 80, I will sit down. This little bit daily, I gradually increased the amount of exercise. It has taken almost a year. Now I can go out twice a day. Go to the park once in the morning, return, and go out once in the afternoon. (P16)	Expansion
Triggers	None	Surroundings and environment: If it’s rainy today, I won’t go out. If it’s a good day, I will go out for a walk. (P22) There is a distance marker in our living room. I will count by myself and walk back and forth when I see it. (P14)	Expansion
	Patients who received professional support after discharge (IRR 8.395, 95% CI 1.349–52.243, *p*=0.023) were more likely to continue maintaining recommended physical activity levels post-TAVR	Professional support: I am willing to listen to the doctor’s or nurse’s advice, and I am willing to learn because the doctor and nurse are more professional. I don’t listen to the fitness trainer, but there is no doctor or nurse to guide me after discharge. (P15) After being discharged, I have been consistently guided by a nurse, who enables me to maintain a positive exercise routine. She is very patient and provides excellent guidance. (P14)	Confirmation/Expansion
	None	Mobile health: I will watch the number of steps on my mobile phone. If I can’t reach the goals I set for myself, I will go right away. (P10)	Expansion
	None	Peer and family support: If I don’t want to exercise sometimes, my wife and children remind me. We also have a WeChat group, where people often remind me to go out for exercise, such as shadowboxing, walking, or doing something. (P23)	Expansion
	Patients engaged in physical activity at a frequency of 3 times or more per week prior to TAVR (IRR 6.933, 95% CI 1.385–34.711, *p*=0.018) were more likely to continue maintaining recommended physical activity levels post-TAVR	Internalization of exercise habits: I have had the habit of exercising since I was a child. I used to do a lot of exercises before, including table tennis, badminton, treadmill, dancing, and playing tai chi circle. (P17)	Confirmation

The qualitative results also revealed other barriers and facilitators to participation in physical activity for patients after TAVR under the guidance of Fogg’s behaviour model. According to Fogg’s behaviour model, 3 elements must converge simultaneously for a behaviour to occur: motivation, ability, and a trigger. When a behaviour does not occur, at least 1 of those 3 elements is missing (32). The barriers and facilitators identified in this study can be used to guide the development of strategies to promote physical activity in patients after TAVR. To help patients maintain recommended physical activity levels after TAVR, enhancing patient motivation, such as through health expectations or social support, ensuring their ability to engage in recommended physical activity through simple steps or home-based exercises, and establishing appropriate conditions to trigger or remind them to exercise, such as using mobile health technologies or providing professional advice, is important. Tailored strategies for motivation promotion, ability assurance, and triggers should be developed on the basis of individual characteristics. Many barriers identified in this study can be addressed through the professional guidance of healthcare providers. In the future, efforts should focus on finding effective ways to ensure continuous health education and guidance for physical activity in patients after TAVR.

### Strengths and limitations

In this study, we used a qualitative approach to complement existing quantitative research, offering a deeper exploration of the intricate factors that act as barriers and facilitators to physical activity among patients post-TAVR. By synthesizing these qualitative findings with the quantitative data from previous studies, our objective was to enhance the comprehensiveness and depth of our understanding of physical activity patterns in this cohort. Limitations include self-reported outcomes potentially introducing information bias, although self-reporting is common in physical activity studies. Additionally, the interviews involved only patients and excluded input from families or stakeholders. Nonetheless, the identified barriers and facilitators at the patient level provide valuable guidance for promoting physical activity.

### Conclusion

A low rate of regular physical activity was found in patients after TAVR. A strategy should be developed to promote physical activity in patients after TAVR, especially those who were physically inactive before TAVR, those who never received guidance on physical activity after discharge, and those who self-reported discomfort after TAVR. The barriers and facilitators at the patient level identified in this study can guide the formulation of measures to promote physical activity in the future.

### Implications for future practice

Medical and rehabilitation staff, including physical therapists, occupational therapists, psychologists, nurses, and other healthcare professionals, play crucial roles in addressing factors contributing to physical inactivity among post-TAVR patients. The findings of this study emphasize the urgent need for their involvement in enhancing post-TAVR patients’ rehabilitation engagement. Moreover, the identified barriers and facilitators to physical activity in post-TAVR patients can be used to guide this multiprofessional team in implementing appropriate measures to promote patients’ physical activity levels. Such measures include prioritizing comprehensive education, tailoring exercise regimens, providing ongoing support, managing symptoms, leveraging peer support, utilizing mobile health technologies, and collaborating with other healthcare professionals. By incorporating these strategies, the multiprofessional rehabilitation team can effectively increase patients’ participation in physical activity and optimize their recovery after TAVR.

## Supplementary Material

BARRIERS AND FACILITATORS TO PHYSICAL ACTIVITY AFTER TRANSCATHETER AORTIC VALVE REPLACEMENT: A MIXED-METHODS STUDY

BARRIERS AND FACILITATORS TO PHYSICAL ACTIVITY AFTER TRANSCATHETER AORTIC VALVE REPLACEMENT: A MIXED-METHODS STUDY
