# Supplementary material for: BARRIERS AND FACILITATORS TO PHYSICAL ACTIVITY AFTER TRANSCATHETER AORTIC VALVE REPLACEMENT: A MIXED-METHODS STUDY
Source: J Rehabil Med. 2025 Feb 18;57:39974. doi: 10.2340/jrm.v57.39974 (PMC11862211; doi:10.2340/jrm.v57.39974)
Supplement: BARRIERS AND FACILITATORS TO PHYSICAL ACTIVITY AFTER TRANSCATHETER AORTIC VALVE REPLACEMENT: A MIXED-METHODS STUDY [file JRM-57-39974-s1.pdf]

Supplementary material has been published as submitted. It has not been copyedited, or typeset by Journal of Rehabilitation Medicine

Table SI. Good Reporting of a Mixed Methods Study (GRAMMS) checklist

Guideline	Section: page
Describe the justification for using a mixed methods approach to the research question	Introduction, Methods-Design
Describe the design in terms of the purpose, priority and sequence of methods	Methods-Design
Describe each method in terms of sampling, data collection and analysis	Methods-Quantitative method, and Qualitative method
Describe where integration has occurred, how it has occurred and who has participated in it	Methods-Data integration<br>Table 4
Describe any limitation of one method associated with the present of the other method	Discussion, Strengths and limitations, Conclusion
Describe any insights gained from mixing or integrating methods	Discussion, Strengths and limitations, Conclusion, Implications for future nursing practice

O'Cathain A, Murphy E, Nicholl J. The quality of mixed methods studies in health services research. J Health Serv Res Policy. 2008;13: 92-98.

Table SII. Representatives of the Coding Processes for Each Category Under the Theme

Codes	Initial concepts	Categories	Theme
"Restoring health is the main objective for me. For my health and to recover better, I must do some exercise. People who do not exercise regularly can't have a healthier body and return to normal life." (P23)<br>"It is mainly to lose weight, but also for health. Exercise is good for the heart and good for the body." (P15)	Exercise to be healthy	Health expectation	Motivation
" Exercise can help me to recover better so that I don't need my family to take care of me. I can go out myself like walking or shopping" (P18)	Exercise to live independently		
"I like to do physical activity in the park, so I can meet many old people and communicate with them." (P14)<br>"I can't just sleep in the bed. Doing physical activity can get me out, and then I can participate in social activities. (P16)	Take part in social activities	Social belonging	Motivation
"Sometimes I go out for a walk in the morning, but my neighbors always say I am not in good health and should not go out frequently. If I want to buy something, they ask my son to buy something for me. My son also doesn't want me to go out walking for a long time. I feel pressured to conform to their advice." (P21)	Seeking validation from others		
"I feel relaxed after exercise. If I don't exercise today, I will feel uncomfortable. I insist on proper exercise so that I can feel more comfortable." (P18)	Feel relaxed after exercise	Feelings after physical activity	Motivation
"I cannot keep exercising now because when I get up in the morning, my feet seem to be very sore. Then my heart beats fast when I move. It's uncomfortable, and I have to block it out. But when I went to the hospital for examination, the doctor said that my heart function was OK and I could exercise. However, every time I exercise, my heart beats fast, and I feel uncomfortable. I just don't want to exercise." (P5)	Feel uncomfortable after exercise		
"Sometimes I fear that too much exercise will reduce the life of our valves." (P16)	Fear that exercise has	Kinesiophobia	Motivation

"I am also fearful that my valve will fall off or shift after exercise." (P17)	adverse effects on artificial valves		
"I'm afraid that after going out for exercise, I will faint just like before the operation. It is too dangerous for me to fall again." (P6)	Fear that exercise will lead to unexpected events		
"When I went to the cardiac rehabilitation centre, the doctor gave me a prescription of physical activity. It was a rehabilitation gymnastics maned Baduanjin. It was very difficult for me to learn and remember the steps of Baduanjin. I used to walk fast, but the pace was slow. So if I could not keep up with the pace, I might give up." (P8)	Steps of the exercise were complex	Complex forms of physical activity	Ability
"I can't learn the exercised you suggested. I don't want to learn any complex steps. I can't remember what you told me." (P2)	Low adherence to complex exercises		
"I do exercise. For example, I can cook some food for my husband and son. I also go to the neighbour's house to talk with them. These activities are also exercise. I think it's enough for me." (P15)	Think of housework as exercise	Misperceptions	Ability
"I went out to play cards and chat with my friends. These are also exercise." (P20)	Think of leisure time as exercise		
"If there is a friend gathering, my exercise plan today will be put on hold." (P13)	Busy with appointments	Schedule conflicts	Ability
"I do all the housework at home. Sometimes I have no time. When my grandson comes back, I will not go out to cook dinner. At this time, I don't want to go out for a walk. I will cook for my grandson. My family likes to eat the food I cook." (P12)	Busy with housework		
"Doctors and nurses asked me to go to the hospital for cardiac rehabilitation. But the distance was too far so that the transportation cost is too high." (P15)	Long distance and high transportation costs	Traffic and distance	Ability
"Shanghai is too far away, and the doctor told me to go for rehabilitation, which I can't do. If I want to go to Shanghai, I will take the high-speed rail. I can't buy tickets by myself, and the children have no time to accompany me. It's so troublesome." (P6)	Long distance and inconvenient transportation		

"After the operation, I have no strength and can't walk independently. I know it's useless to take medicine. I think about it. One is to eat well, the other is to walk with the control of heart rate. I bring a stool. If the heart rate is below 80, I keep walking slowly. If the heart rate is above 80, I will sit down. This little bit daily, I gradually increased the amount of exercise. It has taken almost a year. Now I can go out twice a day. Go out to the park once in the morning, come back, go out once in the afternoon. Now, I can basically walk quickly more than 6000 steps a day. " (P16)	Adjust the amount of exercise according to heart rate	Self-regulation	Ability
"For example, in the morning, I walked more than 4500 steps. After the lunch I walked another 3000 steps. In the evening, I walked more than 3000 steps, and the total number was approximately 10000. I walk almost every day. My goal is to reach 10000 steps. I can reasonably allocate the number of steps into different time so that I can keep walking." (P14)	Allocate the number of steps into different time periods		
"I don't walk when I feel uncomfortable. If I feel well, I will go out and exercise frequently. I can adjust myself in this way according to my feelings, and I think I can stick to it for a long time." (P10)	Adjust the amount of exercise according to feelings		
"I don't want to go out when it's raining. I also haven't gone out in the past two days when it's cold outside." (P20)<br>"If it's rainy today, I won't go out. If it's a good day, I will go out for a walk." (P22)	Bad weather will hinder physical activity, while good weather will trigger physical activity	Surrounding and environment	Triggers
"There is a distance marker in our living room. I will count by myself and walk back and forth when I see it." (P14)<br>"We have a treadmill downstairs, and if I see it, I will remember to walk on it." (P3)	Surroundings can trigger physical activity		
"I am willing to listen to the doctor's or nurse's advice, and I am willing to learn because the doctor and nurse are more professional. I don't listen to the fitness trainer, but there is no doctor or nurse to guide me after discharge." (P15)	Will to listen to professional staff but there is no professional guidance	Professional support	Triggers

"At that time, no one told me how to exercise in the hospital or after discharge. If they did, I would certainly listen to them." (P19)			
"After being discharged, I have been consistently guided by a nurse, who enables me to maintain a positive exercise routine. She is very patient and provide excellent guidance." (P14)	Nurse's professional guidance can help patient to maintain exercise		
"I will watch the number of steps on my mobile phone. If I can't reach the goal, I will go right away." (P10)	Steps on smartphone	Mobile Health	Triggers
"I bought a sports watch. I will deliberately increase my amount of exercise." (P16)	Sports watch		
"If I don't want to exercise sometimes, my wife and my children remind me. We also have a WeChat group, where people often remind me to go out for exercise, such as shadowboxing, walking, or doing something." (P23)	Family and WeChat group	Peer and family support	Triggers
"When my neighbours call me, I will go. When they call me, I will want to go out for a walk." (P18)	Neighbours		
"I have had the habit of exercising since I was a child. I used to do a lot of exercises before, including table tennis, badminton, treadmill, dancing, and playing tai chi circle." (P17)<br>"I have already formed the habit of walking. This is a habit I have always had." (P14)	Previous routine physical activity	Internalization of exercise habits	Triggers
