# Supplementary material for: BARRIERS AND FACILITATORS TO PHYSICAL ACTIVITY AFTER TRANSCATHETER AORTIC VALVE REPLACEMENT: A MIXED-METHODS STUDY
Source: J Rehabil Med. 2025 Feb 18;57:39974. doi: 10.2340/jrm.v57.39974 (PMC11862211; doi:10.2340/jrm.v57.39974)
Supplement: BARRIERS AND FACILITATORS TO PHYSICAL ACTIVITY AFTER TRANSCATHETER AORTIC VALVE REPLACEMENT: A MIXED-METHODS STUDY [file JRM-57-39974-s2.pdf]

Supplementary material has been published as submitted. It has not been copyedited, or typeset by Journal of Rehabilitation Medicine

Appendix S1

The International Physical Activity Questionnaire - Short Form (IPAQ-SF)

We are interested in finding out about the kinds of physical activities that people do as part of their everyday lives. The questions will ask you about the time you spent being physically active **in the last 7 days.** Please answer each question even if you do not consider yourself to be an active person. Please think about the activities you do at work, as part of your house and yard work, to get from place to place, and in your spare time for recreation, exercise or sport.

Think about all the vigorous activities that you did in the **last 7 days. Vigorous** physical activities refer to activities that take hard physical effort and make you breathe much harder than normal. <u>Think only about those physical activities that you did for at least 10 minutes at a time.</u>

1. During the last 7 days, on how many days did you do vigorous physical activities like heavy lifting, digging, aerobics, or fast bicycling?

_____ days per week

☐ No vigorous physical activities (**Skip to question 3)**

2. How much time did you usually spend doing vigorous physical activities on one of those days?

_____ hours per day

_____ minutes per day

☐ Don't know/Not sure

Think about all the moderate activities that you did in the **last 7 days. Moderate** activities refer to activities that take moderate physical effort and make you breathe somewhat harder than normal. <u>Think only about those physical activities that you didfor at least 10 minutes at a time.</u>

3. During the last 7 days, on how many days did you do moderate physical activities like carrying light loads, bicycling at a regular pace, or doubles tennis?

Do not include walking.

_____ days per week

☐ No moderate physical activities **(Skip to question 5)**

4. How much time did you usually spend doing moderate physical activities on one of those days?

_____ hours per day

_____ minutes per day

☐ Don't know/Not sure

Think about the time you spent walking in the last 7 days. This includes at work and at home, walking to travel from place to place, and any other walking that you have done solely for recreation, sport, exercise, or leisure.

5. During the last 7 days, on how many days did you walk for at least 10 minutes at a time?

_____ days per week

☐ No walking **(Skip to question 7)**

6. How much time did you usually spend walking on one of those days?

_____ hours per day

_____ minutes per day

☐ Don't know/Not sure

The last question is about the time you spent sitting on weekdays during the last 7 days. Include time spent at work, at home, while doing course work and during leisure time. This may include time spent sitting at a desk, visiting friends, reading, or sitting or lying down to watch television.

7. During the last 7 days, how much time did you spend sitting on a week day?

_____ hours per day

_____ minutes per day

☐ Don't know/Not sure

This is the end of the questionnaire, thank you for participating

Categories of "physically active" and "physically inactive" obtained with the PAQ-SF

Category	Contents
Physically active	**Correspond to "high" and "moderate" scores of the IPAQ-SF:**<br>**"High PA level"**<br>a) vigorous-intensity PA on ≥ 3 days achieving ≥1500 MET-min/week<br><u>OR</u><br>b) 7 days of any combination of walking, moderate- or vigorous intensity PA achieving ≥3000 MET-min/week<br>**"Moderate PA level"**<br>a) ≥ 3 days of vigorous intensity PA of ≥20 min/ day<br><u>OR</u><br>b) ≥ 5 days of moderate intensity PA and/or walking of ≥30 min/day<br><u>OR</u><br>c) ≥5 days of any combination of walking, moderate- or vigorous intensity PA achieving ≥600 MET-min/week
Physically inactive	**Correspond to "low" score of the IPAQ-SF:**<br>**"Low PA level"**<br>a) No PA is reported<br><u>OR</u><br>b) Some PA is reported but not enough to meet categories "high" or "moderate"

Appendix S2.

The Semistructured Interview Outline:

- How have you performed physical activity since your discharge?
- What motivates you to persist (not persist) in participating in physical activity?
- Why can (can't) you maintain physical activity?
- What factors will trigger you to stay in physical activity?
- What factors will hinder you from maintaining physical activity?
- How do you overcome and solve these barriers during your physical activity?
- What help do you think you need from outside to sustain physical activity?
